# *In-vitro* high-throughput library screening—Kinetics and molecular docking studies of potent inhibitors of α-glucosidase

**DOI:** 10.1371/journal.pone.0286159

**Published:** 2023-06-30

**Authors:** Majid Ali, Khuram Malik, Asma Zaidi, Umar Farooq, Syed Majid Bukhari, Zahid Majeed, Mater H. Mahnashi, Shamyla Nawazish, Alqahtani Abdulwahab, Khaled S. Alshaibari

**Affiliations:** 1 Department of Chemistry, COMSATS University Islamabad, KPK, Abbottabad, Pakistan; 2 Department of Chemistry, Higher Education Department, Government Postgraduate College No.1, Abbottabad, KP, Pakistan; 3 Faculty of Science, Department of Biotechnology, The University of Azad Jammu and Kashmir, Chehla Campus, Muzaffarabad, Pakistan; 4 Department of Pharmaceutical Chemistry, College of Pharmacy, Najran University, Najran, Saudi Arabia; 5 Department of Environmental Sciences, COMSATS University Islamabad, Abbottabad Campus, Abbottabad, Pakistan; 6 Pediatric Department, Medical College, Najran University, Najran, Saudi Arabia; Vignan Pharmacy College, INDIA

## Abstract

High throughput screening of synthetic compounds against vital enzymes is the way forward for the determination of potent enzyme inhibitors. *In-vitro* high throughput library screening of 258 synthetic compounds (comp. **1–258)**, was performed against α-glucosidase. The active compounds out of this library were investigated for their mode of inhibition and binding affinities towards α-glucosidase through kinetics as well as molecular docking studies. Out of all the compounds selected for this study, 63 compounds were found active within the *IC*_50_ range of 3.2 μM to 50.0 μM. The most potent inhibitor of α-glucosidase out of this library was the derivative of an oxadiazole (comp. **25**). It showed the *IC*_50_ value of 3.23 ± 0.8 μM. Other highly active compounds were the derivatives of ethyl-thio benzimidazolyl acetohydrazide with *IC*_50_ values of 6.1 ± 0.5 μM (comp. **228**), 6.84 ± 1.3 μM (comp. **212**), 7.34 ± 0.3 μM (comp. **230**) and 8.93 ± 1.0 μM (comp. **210**). For comparison, the standard (acarbose) showed *IC*_50_ = 378.2 ± 0.12 μM. Kinetic studies of oxadiazole (comp. **25**) and ethylthio benzimidazolyl acetohydrazide (comp. **228**) derivatives indicated that V_max_ and K_m_, both change with changing concentrations of inhibitors which suggests an un-competitive mode of inhibition. Molecular docking studies of these derivatives with the active site of α-glucosidase (PDB ID:1XSK), revealed that these compounds mostly interact with acidic or basic amino acid residues through conventional hydrogen bonds along with other hydrophobic interactions. The binding energy values of compounds **25**, **228**, and **212** were -5.6, -8.7 and -5.4 kcal.mol^-1^ whereas RMSD values were 0.6, 2.0, and 1.7 Å, respectively. For comparison, the co-crystallized ligand showed a binding energy value of -6.6 kcal.mol^-1^ along with an RMSD value of 1.1 Å. Our study predicted several series of compounds as active inhibitors of α-glucosidase including some highly potent inhibitors.

## Introduction

The α-glucosidase is a membrane-bounded enzyme; it is present in the epithelium of the small intestine and is known for its functioning in several metabolic pathways such as carbohydrate digestion, glycolipid, and lyco-protein pathways [1]. The functioning of this enzyme includes the release of α-D-glucose by hydrolysis of non-reducing oligosaccharides and polysaccharides in order to maintain postprandial glucose level [2]. The inhibitors of α-glucosidase possess therapeutic potential against type-2 diabetes mellitus, human immunodeficiency virus infection, obesity, and metastatic cancer [3–5]. There are only three α-glucosidase inhibitors (acarbose, miglitol, and voglibose) that are clinically used today for the treatment of type-2 diabetes. These inhibitors lower the rate of carbohydrate absorption and suppress postprandial hyperglycemia [4]. Furthermore, various studies reported that these inhibitors also possess anticancer, antitumor, and antiviral properties [6]. Oxadiazole derivatives were evaluated against α-glucosidase by Taha Muhammad et al., and he founds several active compounds in the range of *IC*_50_ value of 2.64 ± 0.05 and 460.14 ± 3.25 μM [7]. They also exhibit some other pharmacological activities such as antimicrobial, anticancer, anti-inflammatory, and antioxidant activity [6]. A low micromolar range of anti-α-glucosidase activity by acetohydrazide derivatives were observed by Bekircan, Olcay et al., [8]. Similarly, excellent potency of pyrimidine derivatives with *IC*_50_ values ranging from 16.4 ± 0.36 μM to 297.0 ± 1.2 μM was observed against α-glucosidase by Peytam Fariba et al., [9]. A series of 2-acetyl benzofuran hydrazones and their metal (Zn, Cu, Co, Mn) complexes were screened against inhibitor activity of α-glucosidase by Khan Samra et al., and they observed excellent to moderate activity by these metal complexes [10]. Further design and exploration of new improved α-glucosidase inhibitors are of utmost important for the treatment of related diseases and to eradicate unwanted side effects of commercially available inhibitors.

The present research work is focused on screening a library of (258) synthetic compounds to find potential inhibitors of α-glucosidase. Before this, we recently analyzed and reported almost the same library of compounds against urease and carbonic anhydrase II [11]. Several compounds from oxadiazoles, coumarins, chromane-2,4 diones, and cysteine omeprazole metal complexes were found active against urease. The most promising compound was (R)-1-(4-amino-4-(5-(thiophen-2-yl)-1,3,4-oxadiazol-2-yl)butyl)guanidine with IC50 values of 47 uM. Similarly, some compounds from pyrimidines, oxadiazoles, imidazoles, pyrazoles, hydrazides, and tin-based organic compounds were found active against carbonic anhydrase II. The most active compound was (4-(3-hydroxyphenyl)-6-phenyl-2-thioxo-1,2,3,4-tetrahydropyrimidin-5-yl)phenyl)methanone with IC50 value of 98 uM [11].

## Experimental method

### Chemicals and materials

The chemicals such as the solvents used, enzymes, and respective reagents were acquired from Sigma-Aldrich. The library screening of a total number of 258 synthesized compounds (attained from various sources) was carried out for the determination of their inhibition potential against α-glucosidase. A multichannel pipette was used as a dispenser and a 96-well microplate reader (SpectraMax M2, Molecular Devices, CA, USA) was used for recording inhibition values. The compounds under study had been collected from Organic Synthesis Lab (A-1) and (I-102), Department of Chemistry, COMSATS University Islamabad, Abbottabad Campus, Abbottabad, Pakistan; from the students of Dr. Umar Rashid, Dr. Farhan A. Khan and Dr. Asma Zaidi. The list of different classes of compounds used in this study is given below (Table 1), whereas a complete list of all of the synthetic compounds is given in the S1 Table.

**Table 1:** The list of different classes of synthetic compounds used in this study.

Series #	Name of Series	Comp. #
I	Dinitrophenylhydrazines	**01–15**
II	Oxadiazoles	**16–33**
III	Benzofuran-2-carboxylates	**34–38**
IV	Chromene-2-ones	**39–45**
V	Chromane-2,4-diones	**46–55**
VI	Nitrochromane-2,4-diones	**56–64**
VII	Cysteine-omeprazole metal complexes	**65–74**
VIII	Pyrimidines	**75–87**
IX	Dodecylthio benzimidazolyl acetohydrazides	**88–103**
X	Phenylpiperazine propanamides	**104–109**
XI	Phenylpiperazine benzamides	**110–115**
XII	*N*-substituted benzenesulfonamides (I)	**116–119**
XIII	*N*-substituted benzenesulfonamides (II)	**120–123**
XIV	*N*-substituted benzenesulfonamides (III)	**124–127**
XV	Methoxyphenylsulfonyl 4-methyl triazolyl propanamides	**128–145**
XVI	Methoxyphenylsulfonyl 4-methyl triazolyl piperidines	**146–155**
XVII	Ethylthio benzoimidazolyl methanimines	**156–161**
XVIII	Hexylthio benzimidazolyl acetohydrazides	**162–168**
XIX	Methoxyphenylsulfonyl 4-phenyl triazolyl propanamides	**169–186**
XX	Methoxyphenylsulfonyl 4-phenyl triazolyl piperidines	**187–196**
XXI	Ethylthio benzimidazolyl acetohydrazides	**197–230**
XXII	3-Benzyl 4-phenyl triazoles (I)	**231–241**
XXIII	3-Benzyl 4-phenyl triazoles (II)	**242–252**
XXIV	Dihydroanthracene diones	**253–258**

### Glucosidase inhibition assay

The assay is based on the method given by Pistia-Brueggeman with slight modifications. In a 96-well plate 120 μL of sodium phosphate buffer (*p*H = 7) was taken and 20 μL of the enzyme (2U/mL) was added. Then 20 μL of test compound (0.5 mM) was added and then incubated for 15 min. at 37˚C. In sequence, substrate *p*NPG (20 μL, 1mM) was taken in each well, and dissolved in buffer. The reaction mixture was again incubated for 15 min. at 37˚C. The reaction was stopped by the addition of 20 μL of 0.1 mM Na_2_CO_3_, so that the final volume reaches 200 μL. The activity of the enzyme was measured spectrophotometrically at 405 nm by measuring the concentration of product formed, using SpectraMax M2 (Molecular Devices CA, USA). Each sample was taken in triplicate and the % inhibition was measured.

### Kinetic studies

*IC*_*50*_ values of the most active compounds were calculated by non-linear regression analysis using GraphPad Prism 7.04 and compared with the positive control (Acarbose). For each sample, 4–6 different concentrations were used and analyzed against a single concentration (1 mM) of the substrate (*p*NPG), in a 96-well plate by using the same methodology as described above. The type of enzyme inhibition was determined by using the Line-weaver Burk plot. For this, out of 4 different concentrations of each sample, each concentration is analyzed against 4 different concentrations of substrate ranging from 1 mM to 0.125 mM. The inverse of maximum velocity (1/Vmax), obtained for each concentration, is plotted against the inverse of substrate concentrations (1/[S]) to find out type of enzyme inhibition.

### Molecular docking studies

The highly active compounds were subjected to molecular docking studies using MOE (Molecular Operating Environment) software [12]. Ligands were prepared in ChemDraw Ultra and energy was minimized in MOE. The crystal structure of the enzyme α-glucosidase (PDB ID:1XSK) was retrieved from Protein Data Bank and prepared in MOE through the QuickPrep option [13]. The molecular structures of ligands were docked in the active enzyme pocket with the help of MOE-Dock. Triangle Matcher placement method was used with London dG scoring function. The Induced-Fit method helped in conducting the refinement. Furthermore, the rescoring was carried out by means of Affinity dG. The number of retains was limited to 10 throughout the docking studies. The RMSD values, ligand interactions, and binding energy results were used for the final selection of poses. The three-dimensional docking poses of complexes were obtained with the help of MOE. For obtaining the 2D binding poses of the enzyme-inhibitor complex, the Discovery Studio Visualizer was used [14].

## Results and discussion

### In-vitro high throughput library screening

**Fig 1:**
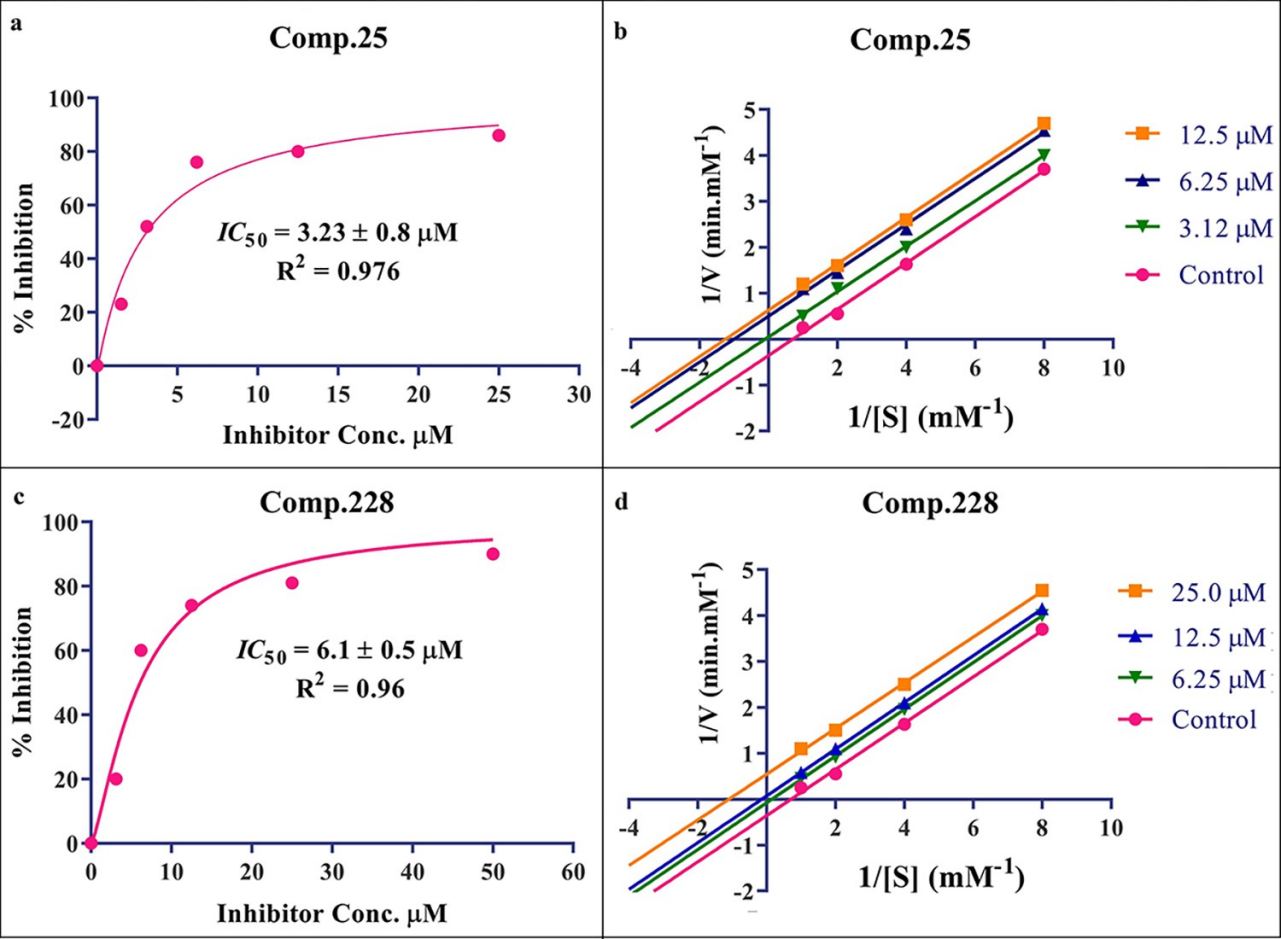
(a) Graph representing *IC*_*50*_ value of compound **25** calculated through non-linear regression analysis, (b) Line-weaver Burk plot for compound **25** representing an un-competitive type of enzyme inhibition, (c) Graph representing *IC*_*50*_ value of compound **228** (d) Line-weaver Burk plot for compound **228** representing an un-competitive type of enzyme inhibition by using GraphPad Prism 7.04.

*In-vitro* high-throughput library screening of 258 synthetic compounds (**1**–**258** –S1 Table, was carried out against α-glucosidase by using a 96-well microplate reader, SpectraMax M2. *IC*_50_ values of the active compounds were determined through GraphPad Prism 7.04 (Fig 1A and 1C).

Overall, 63 compounds were found active within a range of *IC*_50_ value of 3.2 uM to 50 μM (S2 Table), out of which 23 compounds were showing *IC*_50_ values less than 20 μM as provided in Table 2.

**Table 2:** *IC*_*50*_ values of the most active compounds against α-glucosidase.

#	Comp.	Name & Structure	*IC*_*50*_ ± SEM
**1**	**05**	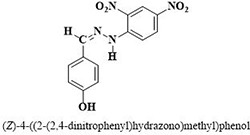	11.3 ± 1.3
**2**	**24**	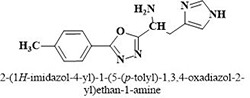	10.82 ± 1.4
**3**	**25**	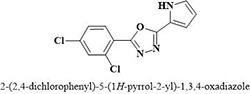	3.23 ± 0.8
**4**	**27**	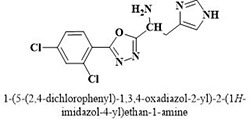	18.32 ± 1.4
**5**	**43**	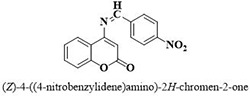	18.8 ± 2.2
**6**	**47**	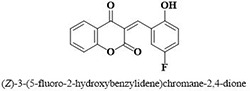	15.2 ± 2.2
**7**	**49**	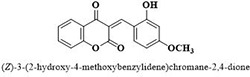	14.1 ± 1.4
**8**	**51**	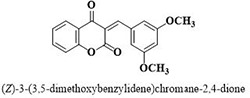	15.9 ± 0.6
**9**	**54**	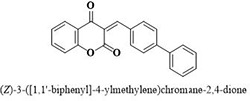	10.5 ± 1.2
**10**	**55**	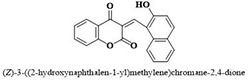	16.1 ± 0.7
**11**	**91**	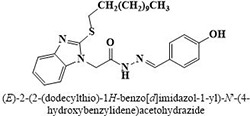	19.8 ± 1.2
**12**	**96**	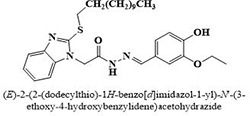	14.7 ± 1.1
**13**	**101**	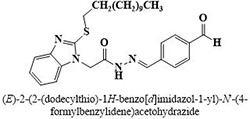	18.2 ± 2.3
**14**	**172**	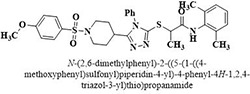	17.2 ± 3.1
**15**	**173**	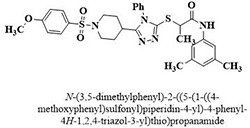	19.4 ± 0.2
**16**	**182**	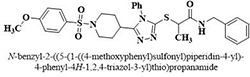	12.3 ± 1.4
**17**	**185**	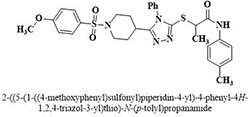	10.9 ± 1.6
**18**	**198**	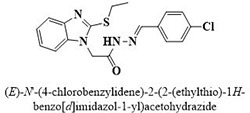	18.7 ± 0.7
**19**	**210**	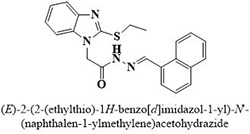	8.93 ± 1.0
**20**	**212**	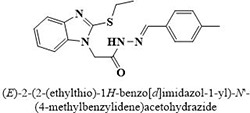	6.84 ± 1.3
**21**	**225**	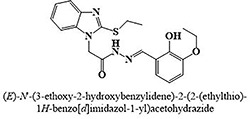	15.8 ± 2.2
**22**	**228**	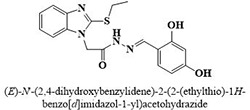	6.10 ± 0.8
**23**	**230**	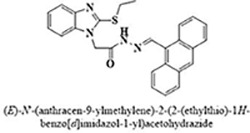	7.34 ± 0.3
**Standard**		**Acarbose**	**378.2 ± 0.12**

Three compounds from series I; dinitrophenylhydrazines **(3**,**5**,**7**), five compounds from series II; oxadiazoles (**24**–**28**), two compounds from series IV; chromene-2-ones (**43**,**45**), nine compounds from series V; chromane-2,4-diones (**46**,**47**,**49**–**55**), sixteen compounds from series IX; dodecylthio benzimidazolyl acetohydrazides (**88**–**103**), eleven compounds from series XIX; methoxyphenylsulfonyl 4-phenyl triazolyl propanamides (**170**–**175**,**182**–**186**), and seventeen compounds from series XXI; ethylthio benzimidazolyl acetohydrazides (**198**,**206**–**208**,**210**,**212**–**218**,**225,228,230**) were found active. Most active compounds with *IC*_50_ values in the range of 3.2 uM to 20 μM are provided in Table 2.

The most potent inhibitor of α-glucosidase was observed from oxadiazole derivatives with *IC*_50_ value of 3.23 ± 0.8 μM (**25**:2-(2,4-dichlorophenyl)-5-(1H-pyrrol-2-yl)-1,3,4-oxadiazole). Other most active compounds were derivatives of ethyl-thio benzimidazolyl acetohydrazides with *IC*_50_ values of 6.10 ± 0.5 μM (**228**:(E)-N’-(2,4-dihydroxybenzylidene)-2-(2-(ethylthio)-1H-benzo[d]imidazol-1-yl)acetohydrazide, 6.84 ± 1.3 μM (**212**:(E)-2-(2-(ethylthio)-1H-benzo[d]imidazol-1-yl)-N’-(4-methylbenzylidene)acetohydrazide), and 7.34 ± 0.3 μM (**230**:(E)-N’-(anthracen-9-ylmethylene)-2-(2-(ethylthio)-1H-benzo[d]imidazol-1-yl)acetohydrazide). In comparison, the standard (acarbose) has *IC*_50_ value = 378.2 ± 0.12 μM [15].

### Kinetic studies

The mode of enzyme inhibition was determined through a Line-weaver Burk plot by using GraphPad Prism 7.04. Kinetic studies of the most active compounds, (**25)** from oxadiazoles (series II) and (**228)** from ethylthio benzimidazolyl acetohydrazides (series XXI), represented that both, V_max,_ and K_m_ changes for each concentration of inhibitor (Fig 1B and 1D) which suggests that they are un-competitive inhibitors of α-glucosidase.

### Molecular docking studies

**Fig 2:**
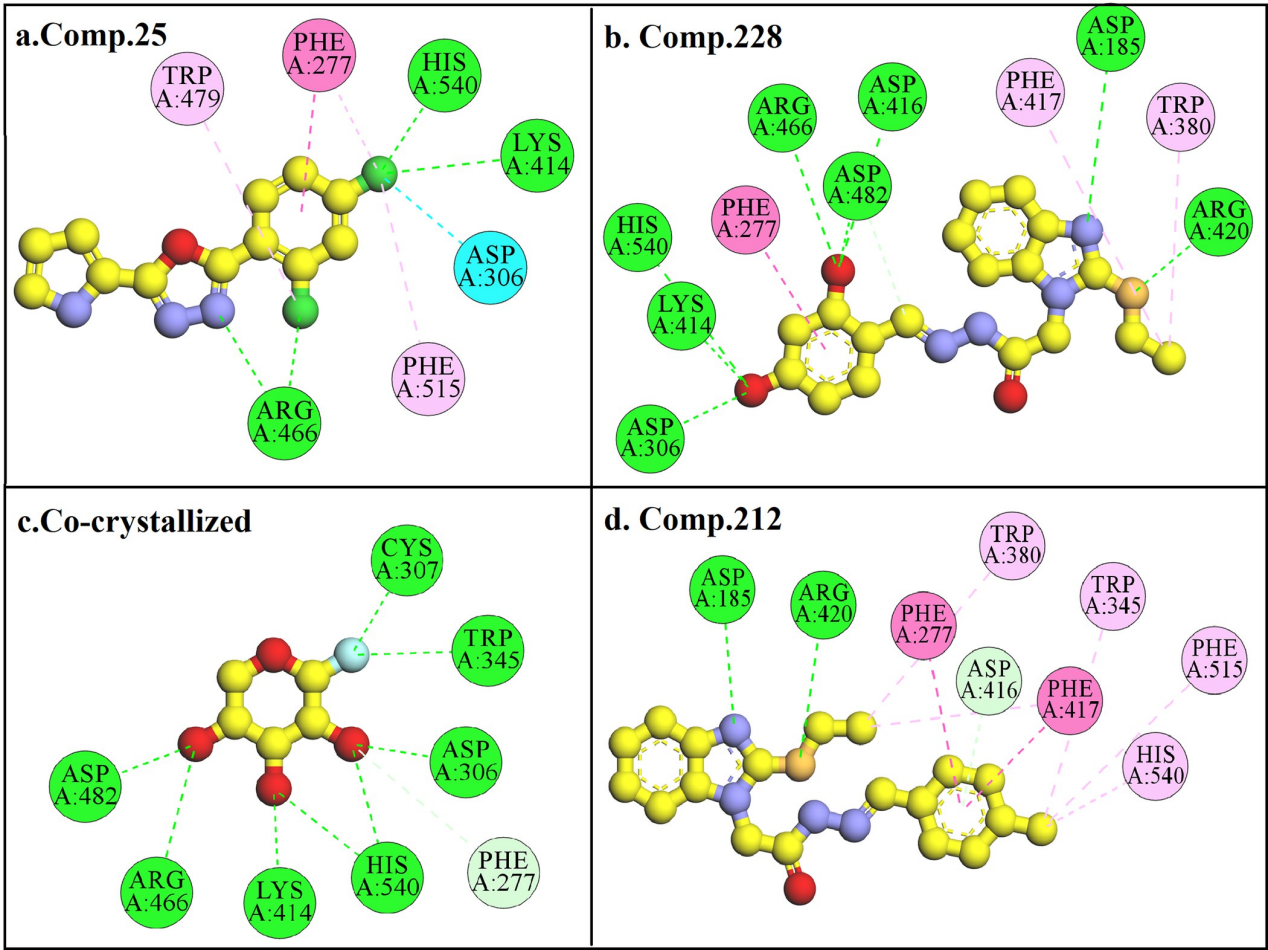
2D, ball and stick model docking pose of compound **25** (a), compound **228** (b), compound **212** (d), and co-crystallized ligand (c). The yellow ball represents carbon atoms, red represents oxygen, blue represents nitrogen, green represents chlorine, brown represents sulfur and cyan represents fluorine. Amino acid residues are shown in the disc model with different colors depending on type of interaction. Green colored disc with dotted lines represents conventional hydrogen bond, dark purple discs represent pi-pi T-shaped interaction, light purple represents pi-alkyl interaction, blue disc with dotted lines represents halogen bond and light blue represents carbon-hydrogen (c) or pi-donor hydrogen (d) bond.Molecular docking studies indicated that compound **25** interacts with Lys414, Arg466, and His540 through conventional hydrogen bond, Phe277 through pi-pi T-shaped, Asp306 through halogen bond, and Trp479 and Phe515 through pi-alkyl interaction as given in Fig 2A. Compound 228 interacts with Asp185, 306, 416, and 482, Arg420 and 466, Lys414, and His54 through conventional hydrogen bonds, Phe277 through pi-pi T-shaped, and Phe417 and Trp380 through pi-alkyl interactions as shown in Fig 2B. Re-docking of co-crystallized ligand indicated conventional hydrogen bonds with Asp306 and 482, Cys307, Trp345, Lys414, Arg466, and His540 as given in Fig 2C. Compound 212 makes conventional hydrogen bonds with Asp184 and Arg420, pi-pi T-shaped with Phe277 and 417, and other pi-alkyl interactions as shown in Fig 2D. According to molecular docking studies, the most active compounds, **25** and **228** have almost similar interactions as compared to the co-crystallized ligand however, compound **212** have slightly different interactions. Structure-activity relationship (SAR), suggests that the activity of the compounds depends on cyclohexane/benzene ring (6-C) with -OH and/or Halogen substitution.

The highly potent compound **25** from oxadiazole derivatives and compounds **228** and **212** from ethyl-thio benzimidazolyl acetohydrazide derivatives were subjected to docking studies in the active pocket of α-glucosidase (PDB ID:1XSK) revealed that these compounds mostly interact with acidic or basic amino acid residues through a conventional hydrogen bond. Binding energy values of compound **25**, **228**, and **212** was -5.6, -8.7, and -5.4 kcal.mol^-1^, and RMSD values of 0.6, 2.0, and 1.7 Å, respectively, which are comparable to the co-crystallized ligand showing binding energy value of -6.6 kcal.mol^-1^ and RMSD value of 1.1 Å. Docking poses of compounds **25**, **228**, **212** and co-crystallized ligand with the active site of α-glucosidase are shown in Fig 2.

## Conclusions

*In-vitro* high throughput library screening of 258 synthetic compounds was carried out against α-glucosidase. Out of which, 63 compounds were found active within a range of *IC*_50_ value of 3.2 μM– 50 μM. The most potent inhibitor of α-glucosidase was the derivative of oxadiazole (comp. **25**: *IC*_50_ = 3.23 ± 0.8 μM) and ethyl-thio benzimidazolyl acetohydrazide (comp. **228**, **212**, **230,** and **210**: *IC*_50_ = 6.10 ± 0.5 μM, 6.84 ± 1.3 μM, 7.34 ± 0.4 μM and 8.93 ± 1.0 μM). Considering the structure-activity relationship of oxadiazole derivatives, most of the compounds were found active containing six-membered or five-membered aromatic rings on both sides of the oxadiazole ring. Most importantly dichlorophenyl ring offers improve activity due to better interaction with the active site cavity of the enzyme, observed by molecular docking studies. Similarly, if we consider the SAR of ethyl-thio- or dodecylthio benzimidazolyl acetohydrazides, the moieties containing substituted benzene rings or fused rings are more active than other compounds, especially the rings containing–OH substitution. Molecular docking studies also indicates the interaction of these–OH groups with the active site residues of the enzyme. Kinetic studies of oxadiazole and ethylthio benzimidazolyl acetohydrazide derivatives indicated an un-competitive mode of inhibition. Our study predicted several potent inhibitors of α-glucosidase.

## Supporting information

S1 Table Library of compounds. (DOCX)

S2 Table *IC*_50_ values of the active compounds against α-glucosidase. (DOCX)

S3 Table Analytical data of most active compounds from top six active classes. (DOCX)
