# Correction: *In-vitro* high-throughput library screening—Kinetics and molecular docking studies of potent inhibitors of α-glucosidase

**DOI:** 10.1371/journal.pone.0294798

**Published:** 2023-11-17

**Authors:** The PLOS ONE Staff

There are errors in the Funding section. The correct Funding statement is: The authors are grateful to the Deanship of Scientific Research, Najran University, Najran, Saudi Arabia for funding this research through research groups grant code NU/RG/MRC/12/3.

The publisher apologizes for the error.
