# Supplementary material for: In-vitro high-throughput library screening—Kinetics and molecular docking studies of potent inhibitors of α-glucosidase
Source: PLoS One. 2023 Jun 30;18(6):e0286159. doi: 10.1371/journal.pone.0286159 (PMC10313066; doi:10.1371/journal.pone.0286159)
Supplement: S1 Table — (DOCX) [file pone.0286159.s001.docx]

**Supplementary table 1. Library of compounds**

| Series I. Dinitrophenylhydrazines | | |
| --- | --- | --- |
| Sr. No. | **Name & Structure** | **Mol. Wt. (g/mol)** |
| 1 |  | 352.1 |
| 2 |  | 344.3 |
| 3 |  | 377.4 |
| 4 |  | 410.4 |
| 5 |  | 317.3 |
| 6 |  | 361.1 |
| 7 |  | 367.3 |
| 8 |  | 370.4 |
| 9 |  | 386.4 |
| 10 |  | 333.3 |
| 11 |  | 346.3 |
| 12 |  | 346.3 |
| 13 |  | 331.3 |
| 14 |  | 225.2 |
| 15 |  | 291.2 |
| Series II. Oxadiazoles | | |
| 16 |  | 288.3 |
| 17 |  | 260.3 |
| 18 |  | 291.3 |
| 19 |  | 325.2 |
| 20 |  | 315.2 |
| 21 |  | 279.3 |
| 22 |  | 310.3 |
| 23 |  | 300.3 |
| 24 |  | 269.1 |
| 25 |  | 279.0 |
| 26 |  | 280.3 |
| 27 |  | 324.2 |
| 28 |  | 290.1 |
| 29 |  | 225.2 |
| 30 |  | 319.3 |
| 31 |  | 334.2 |
| 32 |  | 342.2 |
| 33 |  | 257.2 |
| Series III. Benzofuran-2-carboxylates | | |
| 34 |  | 376.3 |
| 35 |  | 345.4 |
| 36 |  | 365.8 |
| 37 |  | 233.3 |
| 38 |  | 295.3 |
| Series IV. Chromene-2-ones | | |
| 39 |  | 161.2 |
| 40 |  | 283.0 |
| 41 |  | 294.3 |
| 42 |  | 295.3 |
| 43 |  | 294.3 |
| 44 |  | 279.3 |
| 45 |  | 294.3 |
| Series V. Chromane-2,4-diones | | |
| 46 |  | 295.0 |
| 47 |  | 284.0 |
| 48 |  | 350.1 |
| 49 |  | 296.1 |
| 50 |  | 280.1 |
| 51 |  | 310.1 |
| 52 |  | 293.3 |
| 53 |  | 329.1 |
| 54 |  | 326.3 |
| 55 |  | 316.3 |
| Series VI. Nitrochromane-2,4-diones | | |
| 56 |  | 207.0 |
| 57 |  | 340.0 |
| 58 |  | 340.0 |
| 59 |  | 329.0 |
| 60 |  | 325.0 |
| 61 |  | 311.0 |
| 62 |  | 325.0 |
| 63 |  | 311.0 |
| 64 |  | 341.0 |
| Series VII. Cysteine-omeprazole metal complexes | | |
| 65 |  | 530.1 |
| 66 |  | 525.3 |
| 67 |  | 525.5 |
| 68 |  | 521.5 |
| 69 |  | 585.3 |
| 70 |  | 518.6 |
| 71 |  | 522.4 |
| 72 |  | 574.4 |
| 73 |  | 554.2 |
| 74 |  | 560.1 |
| Series VIII. Pyrimidines | | |
| 75 | **** | 274.3 |
| 76 |  | 336.4 |
| 77 |  | 386.5 |
| 78 |  | 259.3 |
| 79 |  | 322.4 |
| 80 |  | 290.3 |
| 81 |  | 357.9 |
| 82 |  | 344.4 |
| 83 |  | 340.9 |
| 84 |  | 295 |
| 85 |  | 276 |
| 86 |  | 246 |
| 87 |  | 259 |
| Series IX. Dodecylthio benzimidazolyl acetohydrazides | | |
| 88 |  | 523.7 |
| 89 |  | 568.8 |
| 90 |  | 513.4 |
| 91 |  | 594.7 |
| 92 |  | 594.7 |
| 93 |  | 508.7 |
| 94 |  | 492.7 |
| 95 |  | 538.7 |
| 96 |  | 538.7 |
| 97 |  | 547.6 |
| 98 |  | 513 |
| 99 |  | 528.7 |
| 100 |  | 549.8 |
| 101 |  | 506.7 |
| 102 |  | 520.7 |
| 103 |  | 524.7 |
| Series X. Phenylpiperazine propanamides | | |
| 104 |  | 353.4 |
| 105 |  | 337.4 |
| 106 |  | 337.4 |
| 107 |  | 337.4 |
| 108 |  | 337.4 |
| 109 |  | 337.4 |
| Series XI. Phenylpiperazine benzamides | | |
| 110 |  | 385.5 |
| 111 |  | 415.5 |
| 112 |  | 399.5 |
| 113 |  | 399.5 |
| 114 |  | 399.5 |
| 115 |  | 385.5 |
| Series XII. *N*-substituted Benzenesulfonamides (I) | | |
| 116 |  | 344.4 |
| 117 |  | 378.9 |
| 118 |  | 413.3 |
| 119 |  | 358.4 |
| Series XIII. *N*-substituted Benzenesulfonamides (II) | | |
| 120 |  | 436.7 |
| 121 |  | 420.3 |
| 122 |  | 354.2 |
| 123 |  | 436.7 |
| Series XIV. *N*-substituted Benzenesulfonamides (III) | | |
| 124 |  | 378.8 |
| 125 |  | 378.9 |
| 126 |  | 413.3 |
| 127 |  | 423.3 |
| Series XV. Methoxyphenylsulfonyl 4-methyl triazolyl propanamides | | |
| 128 |  | 543.7 |
| 129 |  | 543.7 |
| 130 |  | 543.7 |
| 131 |  | 543.7 |
| 132 |  | 543.7 |
| 133 |  | 515.6 |
| 134 |  | 529.7 |
| 135 |  | 529.7 |
| 136 |  | 545.7 |
| 137 |  | 559.7 |
| 138 |  | 557.7 |
| 139 |  | 521.7 |
| 140 |  | 545.7 |
| 141 |  | 529.7 |
| 142 |  | 587.7 |
| 143 |  | 543.7 |
| 144 |  | 529.6 |
| 145 |  | 574.6 |
| Series XVI. Methoxyphenylsulfonyl 4-methyl triazolyl piperidines | | |
| 146 |  | 458.6 |
| 147 |  | 472.6 |
| 148 |  | 472.6 |
| 149 |  | 472.6 |
| 150 |  | 493.0 |
| 151 |  | 493.0 |
| 152 |  | 527.5 |
| 153 |  | 527.5 |
| 154 |  | 537.5 |
| 155 |  | 476.6 |
| Series XVII. Ethylthio benzoimidazolyl methanimines | | |
| 156 |  | 326.4 |
| 157 |  | 327.4 |
| 158 |  | 327.4 |
| 159 |  | 350.3 |
| 160 |  | 297.4 |
| 161 |  | 287.4 |
| Series XVIII. Hexylthio benzoimidazolyl acetohydrazides | | |
| 162 |  | 410.5 |
| 163 |  | 439.5 |
| 164 |  | 484.6 |
| 165 |  | 422.5 |
| 166 |  | 429.0 |
| 167 |  | 454.6 |
| 168 |  | 465.6 |
| Series XIX. Methoxyphenylsulfonyl 4-phenyl triazolyl propanamides | | |
| 169 |  | 605.7 |
| 170 |  | 605.7 |
| 171 |  | 605.7 |
| 172 |  | 605.7 |
| 173 |  | 605.7 |
| 174 |  | 577.8 |
| 175 |  | 591.8 |
| 176 |  | 591.8 |
| 177 |  | 607.8 |
| 178 |  | 621.8 |
| 179 |  | 619.8 |
| 180 |  | 583.7 |
| 181 |  | 607.7 |
| 182 |  | 591.7 |
| 183 |  | 649.8 |
| 184 |  | 605.8 |
| 185 |  | 591.7 |
| 186 |  | 636.7 |
| Series XX. Methoxyphenylsulfonyl 4-phenyl triazolyl piperidines | | |
| 187 |  | 520.6 |
| 188 |  | 534.7 |
| 189 |  | 534.7 |
| 190 |  | 534.7 |
| 191 |  | 555.1 |
| 192 |  | 555.1 |
| 193 |  | 589.6 |
| 194 |  | 589.6 |
| 195 |  | 599.6 |
| 196 |  | 538.6 |
| Series XXI. Ethylthio benzoimidazolyl acetohydrazides | | |
| 197 |  | 354.4 |
| 198 |  | 372.9 |
| 199 |  | 428.5 |
| 200 |  | 407.3 |
| 201 |  | 366.4 |
| 202 |  | 398.5 |
| 203 |  | 354.4 |
| 204 |  | 409.0 |
| 205 |  | 409.5 |
| 206 |  | 384.5 |
| 207 |  | 384.5 |
| 208 |  | 372.9 |
| 209 |  | 398.5 |
| 210 |  | 388.5 |
| 211 |  | 368.5 |
| 212 |  | 352.5 |
| 213 |  | 354.4 |
| 214 |  | 360.5 |
| 215 |  | 342.4 |
| 216 |  | 368.5 |
| 217 |  | 344.5 |
| 218 |  | 380.5 |
| 219 |  | 304.4 |
| 220 |  | 356.4 |
| 221 |  | 328.4 |
| 222 |  | 368.4 |
| 223 |  | 383.4 |
| 224 |  | 417.3 |
| 225 |  | 398.5 |
| 226 |  | 386.4 |
| 227 |  | 370.4 |
| 228 |  | 370.4 |
| 229 |  | 352.5 |
| 230 |  | 438.5 |
| Series XXII. 3-Benzyl 4-phenyl triazoles (I) | | |
| 231 |  | 369 |
| 232 |  | 369 |
| 233 |  | 369 |
| 234 |  | 355 |
| 235 |  | 391.5 |
| 236 |  | 391.5 |
| 237 |  | 427 |
| 238 |  | 427 |
| 239 |  | 375 |
| 240 |  | 435 |
| 241 |  | 385 |
| Series XXIII. 3-Benzyl 4-phenyl triazoles (II) | | |
| 242 |  | 337 |
| 243 |  | 309 |
| 244 |  | 336 |
| 245 |  | 373 |
| 246 |  | 329.5 |
| 247 |  | 295 |
| 248 |  | 323 |
| 249 |  | 309 |
| 250 |  | 337 |
| 251 |  | 309 |
| 252 |  | 353 |
| Series XXIV. Dihydroanthracene diones | | |
| 253 |  | 254.3 |
| 254 |  | 284.3 |
| 255 |  | 270.3 |
| 256 |  | 284.3 |
| 257 |  | 300.1 |
| 258 |  | 268.3 |
