# Supplementary material for: In-vitro high-throughput library screening—Kinetics and molecular docking studies of potent inhibitors of α-glucosidase
Source: PLoS One. 2023 Jun 30;18(6):e0286159. doi: 10.1371/journal.pone.0286159 (PMC10313066; doi:10.1371/journal.pone.0286159)
Supplement: S2 Table — (DOCX) [file pone.0286159.s002.docx]

**Supplementary table 2. *IC*_50_ values of the active compounds against α-glucosidase**

| **#** | **Comp.** | **Name & Structure** | ***IC_50_* ± SEM** |
| --- | --- | --- | --- |
|  | | **Series I. Dinitrophenylhydrazines** |  |
| 1 | **03** | **** | **22.2 ± 1.4** |
| 2 | **05** | **** | **11.3 ± 1.3** |
| 3 | **07** | **** | **26.7 ± 1.6** |
|  | | **Series II. Oxadiazoles** |  |
| 4 | **24** | **** | **10.82 ± 1.4** |
| 5 | **25** | **** | **3.23 ± 0.8** |
| 6 | **26** | **** | **35.30 ± 1.2** |
| 7 | **27** | **** | **18.32 ± 1.4** |
| 8 | **28** | **** | **28.43 ± 2.4** |
|  | | **Series IV. Chromene-2-ones** |  |
| 9 | **43** | **** | **18.8 ± 2.2** |
| 10 | **45** |  | **25.5 ± 2.7** |
|  | | **Series V. Chromane-2,4-diones** |  |
| 11 | **46** | **** | **23.7 ± 1.1** |
| 12 | **47** | **** | **15.2 ± 2.2** |
| 13 | **49** |  | **14.1 ± 1.4** |
| 14 | **50** | **** | **22.4 ± 0.3** |
| 15 | **51** | **** | **15.9 ± 0.6** |
| 16 | **52** | **** | **43.1± 1.2** |
| 17 | **53** | **** | **24.8 ± 0.4** |
| 18 | **54** | **** | **10.5 ± 1.2** |
| 19 | **55** | **** | **16.1 ± 0.7** |
|  | | Series IX. Dodecylthio benzimidazolyl acetohydrazides |  |
| 20 | **88** | **** | **34.8 ± 1.2** |
| 21 | **89** |  | **22.71 ± 1.0** |
| 22 | **90** | **** | **25.20 ± 1.1** |
| 23 | **91** | **** | **19.80 ± 1.2** |
| 24 | **92** | **** | **33.9 ± 0.9** |
| 25 | **93** | **** | **22.4 ± 1.1** |
| 26 | **94** | **** | **25.1 ± 0.8** |
| 27 | **95** | **** | **46.26 ± 0.9** |
| 28 | **96** | **** | **14.7 ± 1.1** |
| 29 | **97** | **** | **48.94 ± 1.3** |
| 30 | **98** | **** | **43.10 ± 1.4** |
| 31 | **99** | **** | **39.01 ± 0.7** |
| 32 | **100** | **** | **47.06 ± 0.8** |
| 33 | **101** |  | **18.20 ± 2.3** |
| 34 | **102** | **** | **49.58 ± 1.1** |
| 35 | **103** | **** | **22.7 ± 1.0** |
|  | | **Series XIX. Methoxyphenylsulfonyl 4-phenyl triazolyl propanamides** |  |
| 36 | **170** | **** | **35.62 ± 2.0** |
| 37 | **171** | **** | **26.01 ± 1.6** |
| 38 | **172** | **** | **17.20 ± 3.1** |
| 39 | **173** | **** | **19.40 ± 0.2** |
| 40 | **174** | **** | **20.6 ± 1.2** |
| 41 | **175** | **** | **46.30 ± 0.4** |
| 42 | **182** | **** | **12.30 ± 1.4** |
| 43 | **183** | **** | **38.55 ± 0.6** |
| 44 | **184** | **** | **36.16 ± 2.1** |
| 45 | **185** | **** | **10.90 ± 1.6** |
| 46 | **186** | **** | **29.01 ± 2.2** |
|  | | **Series XXI. Ethylthio benzoimidazolyl acetohydrazides** |  |
| 47 | **198** |  | **18.70 ± 0.7** |
| 48 | **206** |  | **43.82 ± 0.4** |
| 49 | **207** |  | **36.76 ±1.2** |
| 50 | **208** |  | **29.30 ± 2.0** |
| 51 | **210** |  | **8.93 ± 1.0** |
| 52 | **212** |  | **6.84 ± 1.3** |
| 53 | **213** |  | **22.1 ± 0.2** |
| 54 | **214** |  | **37.04 ± 0.5** |
| 55 | **215** |  | **39.68 ±1.1** |
| 56 | **216** |  | **48.63 ±1.2** |
| 57 | **217** |  | **43.45 ±1.9** |
| 58 | **218** |  | **21.50 ± 2.0** |
| 59 | **220** |  | **24.90 ± 0.3** |
| 60 | **223** |  | **38.03 ± 0.8** |
| 61 | **225** |  | **15.80 ± 2.2** |
| 62 | **228** |  | **6.14 ± 0.8** |
| 63 | **230** |  | **7.34 ± 0.4** |
