# Supplementary material for: In-vitro high-throughput library screening—Kinetics and molecular docking studies of potent inhibitors of α-glucosidase
Source: PLoS One. 2023 Jun 30;18(6):e0286159. doi: 10.1371/journal.pone.0286159 (PMC10313066; doi:10.1371/journal.pone.0286159)
Supplement: S3 Table — (DOCX) [file pone.0286159.s003.docx]

**Supplementary table 3. Analytical data of most active compounds from top six active classes**

| **#** | **Series of compound** | **Compound #** | **OD of sample** | **OD of Control** | **Percentage Inhibition** |
| --- | --- | --- | --- | --- | --- |
| 1 | Oxadiazoles | 25 | 0.33 | 3.30 | 90.0% |
| 2 | Ethylthiobenzimidazolyl acetohydrazide | 228 | 0.30 | 3.43 | 91.2% |
| 3 | Dodecylthiobenzimidazolyl  acetohydrazide | 96 | 0.64 | 3.22 | 80.1% |
| 4 | Chromane-2,4-dione | 54 | 0.39 | 2.71 | 85.6% |
| 5 | Methoxyphenylsulfonyl 4-phenyl triazolyl propanamides | 185 | 0.49 | 2.72 | 81.9% |
| 6 | Dnitrophenyl hydrazine | 05 | 0.62 | 3.20 | 80.6% |
